# A systematic review on status of lead pollution and toxicity in Iran; Guidance for preventive measures

**DOI:** 10.1186/1560-8115-20-2

**Published:** 2012-07-19

**Authors:** Parissa Karrari, Omid Mehrpour, Mohammad Abdollahi

**Affiliations:** 1 Medical Toxicology and Drug Abuse Research Center (MTDRC), Pasdaran Avenue, Birjand University of Medical Sciences, Birjand, 9713643138, Iran; 2 Department of Clinical Toxicology and Forensic Medicine, Faculty of Medicine, Birjand University of Medical Sciences, Ghaffari Avenue, Birjand, 9717853577, Iran; 3 Department of Toxicology and Pharmacology, Faculty of Pharmacy, and Pharmaceutical Sciences Research Center, Tehran University of Medical Sciences, Tehran, 1417614411, Iran

**Keywords:** Iran, Lead, Poisoning, Pollution, Toxicity

## Abstract

Lead is an old environmental metal which is presented everywhere and lead poisoning is an important health issue in many countries in the world including Iran. It is known as a silent environmental disease which can have life-long adverse health effects. In children, the most vulnerable population, mental development of children health effects is of the greatest influence. Low level lead exposure can significantly induce motor dysfunctions and cognitive impairment in children. The sources of lead exposure vary among countries. Occupational lead exposure is an important health issue in Iran and mine workers, employees of paint factories, workers of copying centers, drivers, and tile making factories are in higher risk of lead toxicity. Moreover lead processing industry has always been a major of concern which affects surface water, drinking waters, and ground waters, even water of Caspian Sea, Persian Gulf and rivers due to increasing the number of industries in vicinity of rivers that release their waste discharges into river or sea. In addition, lead contamination of soil and air especially in vicinity of polluted and industrialized cities is another health problem in Iran. Even foods such as rice and fishes, raw milk, and vegetables which are the most common food of Iranian population are polluted to lead in some area of Iran. Adding lead to the opium is a recently health hazard in Iran that has been observed among opium addicts. There are few studies evaluated current status of lead exposure and toxicity in the Iranian children and pregnant women which should be taken into account of authorities. We recommend to identify sources, eliminate or control sources, and monitor environmental exposures and hazards to prevent lead poisoning.

## Review

### Background

Lead is an old environmental xenobiotic metal which is presented everywhere [1] and its chemical properties make a wide spectrum of applications possible for lead. Lead is used in more than 900 industries, including mining, smelting, refining, battery manufacturing and so on [2]. It is one of the most abundant natural substances [3] and is the fifth highest metal used throughout the world. In Iran, application of lead dates back to 5,000 years ago [4] and previous Iranian scientific such as Haly abba (10 th century), Rhazea (865–952 CE) knew about concept of lead poisoning. Iranian people used lead for different purposes such as facial powder, painting, and traditional tile brick glazing. Industries such as mining had not been modernly managed until 1930, thus there were many lead exposure in the workers. Lead toxicity is of the major concerns to public health due to widespread persistence of lead in the environment [5]. In the history of medicine; lead poisoning has been a well-known disease. The first article about lead poisoning was published in 1848 [4]. Although lead toxicity has been relatively controlled in industries but it is still the most common environmental toxicity in the United States of America (U. S.)[6] and it is an important health issue in countries such as Iran [7]. Over the past decades; efforts have been made to reduce its exposure [5]. The activity related to the workers safety and occupational health has been started from 1946 in Iran [4].

Signs and symptoms of lead poisoning included hearing loss, anemia, renal failure, and weakened immune system, and Low birth weights, still births and miscarriages, premature births, and increased urine and blood lead levels (BLL) are the most common reports [8].

BLL provides the best parameter of recent exposure to this metal [9]. Normal BLL is less than 30 μg/dL, whereas acceptable BLL ranges between 30 and 49.9 μg/dl while high BLL refers to higher than 49. 9 μg/dL [9]. The World Health Organization (WHO) expresses the limit for BLL as 1.9 μmol/L (40 μg/dL) for men, and 1.4 μmol/L (30 μg/dL) for women of child-bearing age [3].

There are so many papers about hazards and adverse effects of lead on human worldwide and meanwhile the incidence, prevalence and sources of its contamination is clear. But this is the first comprehensive review about lead toxicity in Iran. The fact is that information on lead contamination in Iran are incomplete and dispersed. In this review, all papers published about sources and hazards of lead exposure in Iran during the past two decades have been gathered and criticized to reach a conclusion about exact risk of this metal in a large country with highest rate of import and export in the Middle East.

## Methods

We looked up the terms lead, Pb, toxicity, poisoning, exposure, source and Iran in all bibliographical databases such as TUMS digital library, PubMed, Scopus and Google Scholar. This review includes relevant articles published between 1990 and 2011.

## Findings and discussions

### Occupational lead

Lead is a toxic heavy metal for human that is recognized as an environmental and occupational hazard. However, in industry, it is a useful metal and is still being used in various industries in Iran, for example, in producing of lead bullets, in battery manufacturing, lead refinery industry, and is used as a smelter metal for purifying gold and silver. The workers who work in these factories can be easily exposed to the dusts or fumes of lead. Occupational lead poisoning has been a human health hazard for more than two centuries [10]. While, acute lead poisoning is rare, subacute and chronic intoxication (occupational) are not uncommon in cities where industries or mines are located. As reviewed by Mañay et al.(2008) in Uruguay, it was revealed that exposed workers with lead from different manufacturing industries such as battery factories, foundries, wire factories, etc., showed that almost 60% of BLL of tested cases were above 40 μg/dL [11]. It has been reported that lead exposure in Brazil occurs mainly in battery plants (recycling plants and lead-acid battery producing). Also lead in pigments, plastic and ceramics, and rubber industries are other concerns in Brazil. Moreover, small recovery battery workshops and medium size secondary smelting plants have been found responsible for the most occupational lead poisoning cases in the Brazil [12]. Similarly, it has been reported that almost 95% of lead poisoning among US adults comes from occupational exposure [7].

In Iran, people or workers of industrial cities such as Tehran, Mashhad, Isfahan, Tabriz, Zanjan, and Arak are at greater risks. Some years ago when leaded gasoline was highly used in Iran, professional drivers such as taxi drivers or bus drivers were found highly-exposed through polluted air [13] but this is not the case in the most recent years. Lead toxicity in drivers may be influenced by availability of lead dust, and spreading in air breathing [3]. Thus, considering the similar countries in the world, people of cities with major motor vehicle traffic and air pollution may be still at risk of lead pollution [13].

Another reported occupational lead exposure is workers of copying centers [9] and employees of paint production factories [3,14]. Inorganic lead compounds are widely used in paint and pigment industries [6] and inhalation seems the most probable route of exposure [9] to lead. Besides, lead naphthenate oxalate is also used as a drying agent in the paint [3] and thus painters seems to have higher BLL but it has not been studied in Iran yet.

Kalantary et al. compared the BLL in workers of Zinc melting factory of Dandi Zanjan with healthy men who were living around the factory and found that BLL in factory workers were higher than that of controls [15]. In another study carried out in Zanjan city on the workers of a lead refinery industry and two control groups, the mean concentrations of hair lead in the lead refinery workers (case group), the staff (control group A) and the citizens (control group B) were 131.7 ± 93.4 μg/g, 21.1 ± 13.2 μg/g, and 27.9 ± 14.1 μg/g, respectively. The mean hair lead concentration in the case group was more than normal range (0–30 μg/g). The mean hair lead level in the citizens who used gas vehicles was statistically higher than who had not used it (36.9 ± 12.2 μg/g vs. 16.6 ± 4.9 μg/g)[16].

Tabrizizadeh et al. evaluated the relationship between the prevalence of oral complications and BLL in workers employed in Koushk lead mines from Yazd province and compared BLL with a control group. They found that factory workers had higher BLL than controls and in the meantime neurologic disorder, chronic fatigue, existence of lead line, mucous pigmentation, gingivitis, tongue burning, taste sense reduction and dimethylformamide (DMF) were higher among workers, although the BLL in most of mine workers was in normal limits [17]. Moreover in a study conducted for determination of BLL on workers of lead and zinc mine in Kooshk City, it was revealed that BLL in 45. 7% of workers were more than permissible limit [18]. In another study in mine workers exposed to lead and zinc in Arak city, it was shown that the mean scores of physical complaints, anxiety, and aggression scales were significantly higher in the case group than the control. They concluded that oxidative stress induced by lead results in mental disorders and thus mine workers suffered from more psychological disorders should be in greater care [19]. Moreover, evaluating BLL in Welders of a car company in suburb of Tehran revealed that BLL in those who smoke more than seven cigarettes per day was significantly higher than those who smoke less than seven cigarettes per day or no smoking group, also the hemoglobin concentrations in frequently cigarette users was significantly lower than that of the non-smokers or less cigarette users. They concluded, cigarette smoking in occupationally lead-exposed workers makes them in higher risk of lead as well as inhibition of hemoglobin synthesis [20]. In another study, lead concentration in urine of urban service workers of Tehran were compared with control group, and the results showed that lead levels in 77. 1% of the urine samples were higher than Health and Safety Executive (HSE)-recommended limits (643.86 ± 353.73). Also, mean urine lead levels in smokers were significantly higher than that of non-smokers in case group [21]. Yartirah et al. in a study on workers of refinery in Kermanshah found that those workers had higher blood and urine lead levels in comparison to control group. Also lead concentration among those who worked with tin was higher than others. In the meantime, there was a correlation between increase of lead level and increase of age or cigarette smoking [22]. Actually, workers who are involved in glazing the traditional tiles are easily exposed to the lead. In a study conducted by Balali-Mood et al. in Mashhad to determine the prevalence of lead intoxication and its complication in traditional tile workers, they concluded that lead toxicity in these workers is not uncommon and the toxic effects of lead were more often found on the teeth, central nervous system and peripheral nervous system [7,23]. Lead is also a significant occupational hazard in ceramic industries and it is still used in ceramic industries in many of Asian countries. Lead glaze is commonly used for hand-crafted pottery in Iran to produce certain colors and help to prevent cracking. Inhalation of airborne lead and ingestion of lead through contaminated hands are generally the common sources of lead absorption in lead-glazed ceramics workers. In Iran, the Lalejin city in Hamadan province is the main center of hand-crafted pottery. This city has about 15,000 residents and about a hundred glazing workshops. Lead, copper, zinc and magnesium are used in these glazing workshops without preventive measures against heavy metal toxicity and the workers of these workshops are at great risk of lead toxicity. Some reports documented lead poisoning coming from these potteries glazing [24].

It has been pointed out that tetraethyl lead is added to petrol for reducing flammability so gasoline station workers are another group at risk of lead toxicity [25]. Repetitive stopping of numerous vehicles, that are coming and going along the days, contaminated floor in gasoline station and workers clothes, makes this group exposed to the lead [26].

It should be considered that battery plant workers, solder ammunitions, workers involved welding and tile making factories, painters, car radiators, manufacturing or use of cable and wires, ceramic ware with lead glaze and tin cans, lead smelting plants and steel plants are other main jobs with possible occupational lead toxicity [2,7,14].

Beside this, workers of battery manufactories were found at great risk of lead toxicity in developing countries such as Uruguay and Brazil [11,12]. In a study performed on 105 workers who were exposed to lead in a car battery manufacturer in Mashhad, Iran, in 2006, it was revealed that all of these workers had lead intoxication with mean BLL of 32.2 ± 13.7 μg/dl [27].

In addition to this, inhalation of fumes from burned car batteries, and ingestion of flaking paint are other occupational sources of lead poising [28].

Direct contact of oral mucosa with the lead in breathing air [17] or difficulty of environment and work conditions as risk factors, and smoking [20] may be reasons of lead toxicity in these workers and more important of that is work location. Of course, age, duration of employment and smoking habit [21,22] have direct effect in toxicity.

### Air as a source of lead exposure

Composition of settled dust is similar to air suspended particulates, so it can be a marker of pollutants such as heavy metal contamination in the air. In China, heavy metals were determined in dust of roads, tunnels, urban parks, playgrounds, children’s nurseries and households [29]. Humans can be exposed to heavy metals such as lead in dust through several routes including inhalation, ingestion and skin. In dusty environments, it is estimated that adults could ingest up to 100 mg dust every day. Children are even exposed to greater amounts of dust than adults due to play behavior and hand-mouth pathway [30]. Exposure to lead for general population comes mainly from airborne dusts containing lead particles and from food or water contaminated by lead, of which 15–30% is inhaled and 70–86% is ingested [13]. Each year, 200 million tons of man-made waste products are released into the air, and 50% of this data belong to burning internal engine [31] and the main reason is due to leaded gasoline that is still used as an automobile fuel [13].

Tehran is one of the crowded cities of the world with vehicular terrific, which leaded gasoline is still used although prohibited. Beside from geographical aspect, Tehran is surrounded on three sides by mountains, with no continuous flow of air, and makes this city the highest polluted area [13]. Six types of materials have been known as the major air pollutants which make more than 90% of air pollutants. These include carbon monoxide, nitrogen oxide, hydrocarbons, sulfur oxide, suspended solids, and lead. Those who live in south and central part of Tehran had the highest BLL [32] and those who live in downtown and busy streets are in higher risk of lead toxicity [13] in comparison to those who live in suburb. In a study conducted by Farzin et al. (2008) for obtaining the usual value of Pb, Cd, and Hg in normal human blood of 101 volunteers resident in Tehran, it was found that BLL in normal volunteers living in Tehran were 123.75 ± 56.42 and their results showed significantly higher content of Pb in blood of males compared to females (138.11 ± 65.43 and 101.84 ± 51.38 μg/dL, respectively)[33].

Another study in Kerman one of industrial cities in center of Iran on lead concentration of gasoline station air indicated that the mean value of all the stations was higher than the control one, but because of lack of inversion phenomenon in this city, lead concentration in all the gasoline stations was lower than the leads TLV (total lead value) [26].

High population, vehicles, urban activities, and around industries have made Yazd a city in center of Iran to face serious air lead pollution [34]. It has been estimated that 60–90% of lead in airborne dust of the ambient and 10–50% of lead in the blood of the non-occupationally-lead-exposed population can be attributed to lead in gasoline [13]. Evaluation of suspended air particles and their composition in central area of this city showed that it is higher than national standard [34].

Also it has been reported that air of Zanjan a city in which major lead and zinc factories are located its around is full of heavy metals [35]. Beside, in a study conducted in Tehran, about 40% of randomly selected children had higher BLL which clearly showed importance of screening test for lead poisoning in the population [36]. The measurement of heavy metals in atmospheric precipitation shows the effects of anthropogenic sources in air quality. The heavy metals concentration can be used as air pollution index [24]. Some European countries such as Bulgaria were lucky in full banning import and use of leaded gasoline. The lead levels in Varna, the third largest city, decreased up to 63-fold in year of 1996–2007 [37]. It seems it is the time to be so serious in policies to decrease heavy metal air pollution in Iran such as full prohibition of using leaded gasoline and advises to use filters to reduce the amount of pollutants produced in factories, relocating factories to outside of cities, getting rid of old automobiles and regulating automobile engines that all are undergoing.

### Water as a source of lead exposure

Heavy metals processing industry has always been a major of concern which affect surface water, drinking waters, ground waters and rivers contamination. There are several sources of water around us which make concern about our future life. All of these sources should be examined for the presence of lead when determining a person’s total lead exposure and risk.

Based on WHO standard, concentration of lead in drinking water was limited to 0.01 mg/L, and based on drinking water standard in Iran, upper limit of the concentration of lead in drinking water announced to 0.05 mg/L [38,39]. Lead exposure from drinking water has been a topic of public prevention programs in European countries [40]. To assess the present state of drinking water contamination with lead, a free examination of lead in drinking water was offered in cooperation with local public health departments for private households in Germany. In the screening part of that project, 2,901 tap water collected during 2005–2007 which of those, 7.5% had lead concentration of more than 10 μg/L (recommended limit of the WHO) and 3.3% had concentrations above the present limit of the German drinking water ordinance (25 μg/L)[40].

Recently the problem received attention in the US when report of drinking water at schools was published [41]. Beside, several European countries are known to have significant numbers of building with elevated concentration of lead in drinking water, such as UK, Austria, and Germany [42-44]. In a study conducted in UK (1996), it was found that 17% of households had water lead concentration of 10 μg/L (48. 3 nmol/L) or more in 1993 in comparison with 49% of households in 1981. Meanwhile, tap water lead remained the main cause of raised maternal BLL accounted for 62% and 76% of cases whom maternal BLL were above 5 and 10 μg/dL (0. 24 and 0. 48 μmol/L), respectively [42].

In another study conducted in Austria (2002), the collecting data of the upper floors showed significantly higher lead concentration compared to the lower floors, which indicates that in Viennese drinking water, house installations were the major causes of lead contamination, but in comparison to other European countries the percentage of samples exorbitant the guideline levels (50 μg/L as current value and 10 μg/L as target value) was lower.

Typically, lead gets into tap water after it leaves the water treatment plant, so its monitoring is difficult and somehow impossible to estimate such exposures to lead and other metals, because contamination occurs when the distribution system is not monitored [43].

Lead contamination of drinking water is also a major concern in Iran. In a study carried out to determine heavy metals in water sources of Hamadan city (West of Iran) in 1994, 90 water samples were analyzed and the results showed that the mean concentration of lead were 0. 514 mg/L, which are higher than the standard levels and the authors concluded that these pollutants are mainly sourced from industrial waste and/or fuel consumption. Authors suggested authorities to force factories to restructure their wastewater treatment plant [45].

Another study conducted in Ahwaz city (South-West of Iran) to evaluate the corrosion and leakage potentials of some important heavy metals (Pb, Cd, Zn, Cu, Fe, Mn) using the USEPA (United States Environmental Protection Agency) standard procedure. They selected 76 sampling points including raw water intakes, treatment effluents, and tap waters in Ahwaz distribution network. The results from six rounds of tests showed a lead concentration of 8. 47 μg/L in drinking water. Furthermore, the data indicated high corrosion potential in Ahwaz drinking water distribution network and the leakage of lead and other heavy metals into the network closely associated with the corrosion phenomenon [46].

Shah-Mansouri et al. (2003) evaluated trace metals in the drinking water distribution system in Zarin Shahr and Mobareke of Isfahan province. They found that the average concentration of lead in water distribution system of Zarin Shahr were 5.7 μg/L and in Mobareke were 7.83 μg/L. They discovered that lead concentration was zero at the beginning of the water samples from the municipal drinking water distribution system for both cities. Also they showed corrosion by lead that was the result of dissolution of the galvanized pipes and brass facets. Lead concentration in over 10% of the water samples of Zarin Shahr exceeded the drinking water standard level [47]. In another survey conducted in Isfahan city to evaluate the leakage of heavy metals from the polypropylene pipes and PVC which are used in the water distribution system, the mean lead concentration in old and new PVC pipes was higher than other pipes. Mean leakage of lead was higher in polypropylene (PP) pipes produced in manufacturing plants. Lead leakage was lower than Iranian standards, but exceeds than EPA standards or WHO guidelines in PP pipes produced in manufacturing plants [48]. Thus, it seems use of these types of pipes in the water distribution systems may increase lead concentration in drinking water. In the meantime, metallic structure and inappropriate plastic production are potential factors in contamination of network drinking water with heavy metals. A high protein of lead concentration in municipal drinking water may be related to dissolution of the brass facets and galvanized pipes [47] and leakage of heavy metals from the PVC and PPpipes used in distribution system [48].

Ground water resources in arid and semiarid regions are very important [49]. Groundwater is contaminated by agricultural, industrial and municipal activities [50]. In US, more than half of the consuming water for population and one-third of water for agriculture is supported by groundwater [51]. Thus lead contamination of this groundwater can cause health problems for a country.

Sanitary landfill is one of the potential factors in contamination of ground water. Utilizing the groundwater closed to the landfill and lack of insulating the landfills floor, makes easy movement of current contamination into groundwater. Ebrahimi et al., evaluated pH and metal concentration of area’s groundwater near the municipal solid waste landfill of Yazd city in center of Iran and compared with the ground waters far from them. It was found that the ground waters pH in downstream was significantly lower than upstream and both case and control groundwater were contaminated with lead at the same amount [49]. Moreover in a survey on chemical quality of groundwaters in Zarin Shahr city, it was found that the mean concentration of lead and cadmium exceeded than standard levels. The authors concluded that the water wells are polluted due to high discharge rate of agricultural and industrial wastewater [50].

It is essential to control and treat the wastewater appropriately and also to monitor the groundwater to prevent the aquifer pollution. In another study looking for heavy metals in soils, water, and vegetables of Shahnama region in Shahroud city, it was revealed that mean concentration of lead in water samples were 7. 55 mg/L, which was much higher than standard value (0. 71 mg/L). The authors concluded that use of synthetic fertilizers, unsanitary disposal of sewage and fossil fuel combustion has made water, soil and plants of the region polluted with heavy metals [52].

In the study by Mohammadian et al. (2008), water wells close to Zanjan zinc and lead smelting plant was examined and a lead concentration of 53% in water wells was found that is higher than standard values of WHO [53].

Collection and storage of roof rainwater (Cisterns) in rural areas are traditionally done from long time ago in Iran and many other countries. Many residents in rural areas of Turkaman Sahra located in Golestan province in North of Iran are providing part of drinking and municipal water by this way [54]. In a study detecting probable contamination resources in cisterns in this province, it was found that lead concentration in 51% of samples were higher than reference level. Any of water cisterns in this province was unfavorable for drinking due to lead contamination. Lead pollution in the roof rainwater maybe due to infiltration surface and agricultural waters and precipitation of air pollution and high lead content in these water indicate the need for some form of treatment [54,55].

### Soil as a source of lead exposure

Soil contaminated with lead is not only a major concern in developing countries, but also it is a health problem in western countries. Rabito et al. recently reported the high incidence (61%) of lead above recommended levels in soil and dust samples of New Orleans in the US. Most notably children and around residences were concerned about potential health risks to the lead-contaminated soil and dust in that area [56].

Plant and soil surface are the major sink for airborne lead in the environment and may take a contribution to dietary lead intake [57]. Hereby, in the following paragraphs, we are going to illustrate lead contamination potential of soil in industrial areas, vehicular traffics, and near shore areas.

The rapid industrialization in developing countries frequently causes a high anthropogenic emission of heavy metals into the soil [58]. Hamadan province is located in West of Iran with 1.75 million inhabitants, and semi–arid climate. Although agriculture is a major habit of these people but this city has becoming industrial in the recent years [58]. Jalali et al. researched about contamination of lead in industrial areas in this province. They revealed that industrial soils were contaminated with lead to some extent. Application of sewage sludge, fertilizers, and pesticide in agriculture [58], and industrial activities such as opencast mining and smelting [59], and failure to complete recycling of city refuses or discharge of municipal waste urban in soil had a serious environmental impact on this area and contributed to a continuous accumulation of heavy metals in soil [58].

Zanjan province located in North-West of Iran has been considered as a traditional mining region since ancient time [59] and there are several studies reporting heavy metal contamination in vicinity of lead and zinc mines [53,59,60]. Parizanganeh et al. studied heavy metal pollution in superficial soils surrounding industrial area in Zanjan and found wide spread heavy metal contamination of soil [59]. In another study, soil, water and vegetables of Shahnama region in Shahroud city was examined and the mean concentration of lead in soil was found 81.12 (μg/g), which was much higher than standard value (0.2–1 μg/g). The authors concluded that use of synthetic fertilizers, unsanitary disposal of sewage and fossil fuel combustion has resulted in pollution of water, soil and plants of the region with heavy metals [52].

Pollution caused by traffic activities and exhaust product of leaded gasoline, are one of the major source of contamination by lead in urban environment [57,61]. Expositing to contaminated surface soil with lead, through indoor and outdoor inhalation of lead in dust and ingestion of lead deposited within houses [61]. Farsam et al. studied lead deposition on plant leaves in Tehran and reported that older leaves have higher lead level [57]. In overall, their results tended them to the conclusion that major cause of lead contamination in downtown plants is vehicle exhaust and low rain fall [57]. In another study conducted to investigate the concentration of lead, cadmium, copper and zinc in different sites of the Sari-Ghaemshahr road in Iran [61] which is one of the crowded roads in North of Iran, the soil samples were collected along the sampling section with different distances from the road edge of both sides of the road. Their results showed high amount of lead in nearest distance to the road. Of course, amount of heavy metals is basically dependent on wind [62], traffic intensity, and tire wear [63]. Thus, the highest value of lead in nearest distances could be because of emissions from vehicle exhausts.

In another study, concentration of heavy metals (Pb, AL,Cu, Ni, Zn) in near shore sediments in alongshore direction of the Iranian coast of Caspian Sea was examined [64]. The results showed that concentrations reflected metal loading from anthropogenic sources located at and in the vicinity of the sampling sites [64]. Metal discharged into coastal areas of marine environments is likely to be scavenged by particles and removed to the sediments. So sediments, become large repositories of toxic heavy metals. In overall, although those experiments give information concerning possible enrichment of the soil with heavy metal, but the severity of pollution depends also on the proportion of their mobile and bioavailable form which determines their mobilization capacity and behavior in the environment [58].

### Fish

Heavy metals have a high resistance against degeneration (stable pollution) [65]. Thus, their amount in fish may be increased even several times either in water or air, due to bioaccumulation though fish is often at the top aquatic food chain [8].

Fish as human food is considered as a good source of protein, polyunsaturated fatty acids (omega-3), calcium, iron, zinc and generous supply of minerals and vitamins [66]. The demand for fish as a source of protein is on arise. During the last few decades, great attention has been paid to the possible hazards of heavy metal poisoning in human due to the consumption of contaminated fish. Based on our statistics its consumption during last 20 years, its increased up to 5 kg per capita in Iran [67]. Here we are going to illustrate 4 different parts of Iran that measurement of lead in fish was conducted in the last decade.

#### Caspian Sea

The Caspian Sea with 386,400 Km^2^, with 5 major inlets and no outlet acting as a watershed reservoir is the largest lake surrounded by 5 countries, Azerbaijan, Iran, The Russian Federal, Kazakhstan and Turkmenistan [8]. Determination of lead in the most consumed fishes in Caspian Sea in different studies [8,66,68] revealed existence of exposure to lead.

Shokrzadeh et al. (2004) measured the amount of lead in five species of most consumed fishes in five fishery areas of the Caspian Sea region and their results showed that Rutilus frsii kutum fish had the highest concentration of lead compared to other fishes. Rutilus frsii kutum is living in depth of water, and in this depth, concentration of heavy metals in animals tissue are much higher in comparison to other parts of water [68]. Thus this kind of fish has the most concentration of lead in comparison to the rest. Although lead concentration of Rutilus frsii kutum fish was at standard levels (less than 0.5 ppm) but it was a significant increase in its lead concentration comparing to year of 1997 with value of 0. 07 ppm [68]. Clupeonella delicatulu is another kind of consumed fish from Caspian Sea in North of Iran, and its calcium was replaced with heavy metals from contaminated water because it is consumed with bone, it can cause lead toxicity in humans [68]. Gorgan coast is located in southeastern of Caspian Sea. This coast is one of the most important ecosystems in the North of Iran [66]. Large amount of pesticide and chemical fertilizers containing heavy metals that are used by agricultural industry of this region, are brought via surface run off from farm to river and increases lead concentration in Caspian Sea and its fishes [66]. Tabari et al. (2010) conducted a study on Rutilus frsii kutum, Cyprinus carpio, Mugila auratus in Gorgan coast and reported increasing hazard of lead concentration in fishes, water and the sediment [66]. Also Eslami et al. (2011) measured lead level in Rutilus frisii kutum from Tajan River, one of the significant rivers of Caspian Sea water basin [8] and reported existence of lead toxicity. In above investigations that carried out in one region, it is clear that although the observed heavy metals concentrations were below the recommended limit, but existence of pollution cannot be ignored [66,68]. In Eslami et al. study, they found nearly all non-essential metals (Cd, Ni, Pb) in Rutilus frisii kutum fish higher than limits for fish proposed by FAO/WHO and EU [8].

Besides, Rutilus frisii kutum was the common fish among these studies, this fish is a very valuable commercial fish in that region, with a high demand, due to good taste and kitchen customs, and is consumed in all year around, the average annual catch of Kutum in Iran was about 96,000 Rials in 1991–2001 [69]. In relation to this, spreading lead toxicity through fish and fishery product consumption would be catastrophic.

Increasing the number of factories and industries in vicinity of rivers and receiving effluent discharges which end to sea, presence of thousands of vehicular traffics spreading heavy metals like lead into atmosphere, raining back to earth and sea, leaking petrol from petrol port during exiting or transmission of oil, and land-locked body of water boarded by five countries are all common reasons which makes Caspian sea in priority to any measures to reduce environmental pollution [8,66,68].

### Kor River

The Kor River is the longest freshwater river in Fars Province in South of Iran, it is approximately 50 km long, 15–20 km wide and nearly 20 m depth and it originates from Zagros Mountain. The Kor River is used for irrigation of rice paddies and homesteads, as the supply of drinking water and industrial water needs, and for hydroelectric energy production [67]. Every year the entrance of factory waste such as Shiraz Petrochemical Complex, Marvdasht sugar cube factory, and Charmineh factory and other industrial units into the Kor increase [70]. In a study in Fars province, Ebrahimi et al. [67] studied on lead concentration in (Cyprinus Carpri and Capoeta sp.) from Kor River indicating presence of maximum amount of lead higher than the permissible levels for human consumption [67]. Also it was revealed that lead toxicity in the fishes can induce pathological changes in blood cells, liver and kidney of fishes and these changes were significantly higher in highly polluted area [67].

#### Persian Gulf

The Persian Gulf is an extension of the Arabian Sea, positioned in the heart of the Middle East. It connects with the Gulf of Oman and the Arabian Sea through the Strait of Hormuz, and it’s approximately 990 km long. The Persian Gulf is certainly one of the most vital strategic bodies of water on the planet, as gas and oil from Middle Eastern countries flow through it, supplying most of the world’s energy needs. The Persian Gulf has been subject to inputs of heavy metals from different sources, and it has been estimated that oil contamination in the Persian Gulf represents 4. 7% of the total oil pollution in the world (National Research Council, 1985)[71]. This oil pollution has increased even more after the wars occurred around Persian Gulf, about 11 million oil barrels were discharged into the Persian Gulf [72].

Shahriari et al. [65] reported that in edible tissue of Lutjans Coccineus and Tigeratooh Croaker in Persian Gulf, concentration of lead in 27% of collected samples was more than acceptable limit of WHO [65].

Ashraf in a study evaluated lead level in the kidney and heart tissues of Epinephelus Microdon collected from the Persian Gulf, Eastern province of Saudi Arabia, and it was found that the average lead (3.19 ± 2.03 ppm) concentrations of heart tissues is exactly high [73].

Raissy et al. (2011) in a study determined the concentrations of lead and 3 other toxic metals in lobster (Panulirus homarus) muscles from the Persian Gulf. Lead concentrations in muscle samples were 379–1,120 μg/kg, with means of 629. 4 μg/kg. Lead in the edible muscle tissue, was above the acceptable level and showed a health risk for consumers [74].

#### Farmed fishes

Pourmoghaddas et al. in a study measured lead concentration in Cyprinus carpio (farmed fishes) and Lutjans Coccineus and Tigeratooh Croaker (from Persian Gulf) species which are the most consuming fishes in Isfahan city [75] and found a mean concentration of 0. 48 ppm for lead in the Cyprinus carpio fishes. It was also revealed that lead concentration in 27% of collected cases were more than upper limit in WHO [75]. High lead concentration in farmed fishes maybe due to limited sources of pound water and even there are situations that replace sewage agriculture instead of fresh water in some regions of this city. Also in this study, it was found that lead concentration in the farmed fishes is more than those collected from Persian Gulf. Although lead concentration in Caspian Sea fish in comparison to this region was two times more [75].

Determination of heavy metals in fish in different parts of Iran depends on concentration of the metals in water and exposure period [67], geographical area, quality of water source, distance of industrial units to coast, legal rules in disposal of sewage effluent, type of fish, type of organ tissue, condition of laboratory experimental [65] and other environmental factors, such as salinity, pH, water hardness, and temperature [67].

### Rice

Lead is an unnecessary metal for human body, and any amount of it would be harmful [76] but it is accumulated in rice that is the most popular food among Asian people probably causing silent toxicity demonstrating itself as insufficiency in different tissues and organs [53,77]. Every year, factories and industrial units, and city sewers cause the pollution of agricultural land by adding large resources of contaminated water containing heavy metals [56]. Irrigation of farmland and cropland with this water can cause potential harm for human [70].

The lead content in rice samples from various countries ranged from 1.6 to 58.3 ng/g and the average content was 15.7 ng/g [78].

Jahedkhaniki et al. (2005) determined the lead contents in rice in the North of Iran. They collected samples from four areas of Qaemshahr region in North of Iran (Mazandaran province) at harvesting time of rice. Their results showed that average concentrations of lead in rice was 2.23 ± 18 mg/kg dry weight, which was upper than the FAO/WHO limits. Also the weekly intake of lead from rice was upper than the maximum weekly intake recommended by WHO/FAO [77].

Bakhtiarian et al. (2001) evaluated the effect of the Kor River’s pollution on the lead and cadmium content of the Korbal rice samples in Fars Province in South of Iran [70]. Comparison of the pollution level of the Korbal and Gilan rice samples (which were cultivated with unpolluted water) indicated a significant difference and confirmed the significant effect of the pollution of the river on the lead and cadmium content of the Korbal rice samples. The reason is the entrance of drainage water from different factories like petrochemical factory, charmineh factory, and other industrial units and also entrance of Marvdasht and Zarghan sewer system wastes into the Kor River that is used for cultivation of the rice [70].

Shakerian et al. (2012) investigated the lead content of several commercially available brands of rice grains in central Iran. The results showed that lead concentration in rice grains ranged from 0. 0405 to 0. 1281 ppm dry weight and its average concentration was 0.068 ± 0.0185 ppm. They found that lead concentration in the sampled rice grains was lower in comparison with their upper limits (0. 2 ppm)[79].

In another survey by Malakootian et al. (2011) about determination of lead concentration in imported Indian rice, the result indicated that weekly intake of heavy metal by rice was below the provisional tolerable weekly intake recommended by WHO/FAO [76], but their results were against that of Bakhtiarian et al. who studied in South of Iran and Jahedkhaniki who studied in North of Iran which reported higher lead concentrations in rice samples [70,77]. The most important anthropogenic sources of soil pollution to metal are industrial sludge, effluent discharging, using super phosphate fertilizers, burying the non-ferrous waste in land and closing the agricultural fields to zinc mine and lead or refining factories [80]. These metals accumulate in agricultural products and enter to food chain. In overall health risk of lead intake through rice is high in Iran and might be even increased with consumption of vegetable, fish, etc. [77].

### Vegetables

Food safety is a major public concern worldwide. Vegetables constitute essential components of diet such as vitamins, fiber, mineral, and other nutrients. In a study, determining of heavy metals in soils, water, and vegetables of Shahnama region in Shahroud city indicated that mean concentration of lead in soil samples was 23. 99 (μg/g) that was much higher than standard value (0. 1–10 μg/g). The authors correlated it to use of synthetic fertilizers, unsanitary disposal of sewage and fossil fuel combustion, water, soil and plants of the region that are polluted with heavy metals [52]. Heavy metals such as lead are easily absorbed by soil but have no toxicity for plants [81]. There is evidence suggesting that vegetables cultivation vary in uptake of pollutants [82]. Generally, plants translocate larger quantities of metals into their leaves rather than to their fruits and seed [82]. The amount of lead in the soil is important due to the direct transmission of lead, and also due to forming water-soluble forms of lead by streams of water or rain [82], furthermore the effect of lead in the water or air is directly transmitted [82]. These amounts maybe hazardous if the vegetables are taken in large quantities. Nonetheless, all these metals have toxic potential, but the detrimental impact become apparent only after decades of exposure.

Contamination of vegetables with heavy metals may be due to irrigation with contaminated water [45,52,83-85], addition of fertilizers and metal-based pesticides, industrial emissions, transportation, harvesting process, and storage at the point of sale [83].

There are several studies indicating that irrigation with polluted water is the main source of lead in vegetables [45,83,84]. In another study about determination of lead in cultural vegetables in suburb in Shahroud city, it was shown that surplus water of urban and industrial facilities are main reason of rising lead content to above the standard zone in cultural vegetables [52].

The accumulation of heavy metals varies greatly both between species and cultivars [82]. Lettuce is one of those vegetables that has high capacity in absorption and storage of lead [81] through contaminated soil by sewage or dust deposited on plants exposed to polluted air [81,83]. In Malakootian et al. (2009) study, the mean concentration of lead in lettuce imported to Kerman city from Dezfool, Jahrom, Yazd and Varamin cities were lower than WHO guideline while the lead level in lettuce in Turkey and Kenya was higher than that of used in Kerman [81].

Tea is the most popular beverage in Iran and the presence of lead in tea has been a concern in the recent years. A study by Ebadi et al. (2005) done in Gilan province (North of Iran) on green leaf of tea cultivated in Lahijan and Fuman cities indicated that green leaf of tea in this region had very high amount of lead. In explanation, this region is a transit automobile way for import and export inside the country [82]. In another survey which was carried out on consumed black tea in Tehran, eleven types of the most widely consumed brands of dry black tea were purchased from local market of Tehran, and the results indicated that lead concentration in black tea was higher than the permissible limit for human food [86] keeping in mind that tea is not always used in the fresh green leaf like other vegetables. Matsuura et al. reported that after making tea, 80% of lead content is reduced in comparison to dry tea [87].

### Raw milk

Milk is one of the important selective foods to nourish infant and other age groups. Many reports indicate the presence of heavy metals in milk [88,89]. In Tajkarimi et al study that lead residue in raw milk from different parts of Iran was assessed, the cities of Isfahan, Tehran, and West Azerbaijan showed higher levels of contamination [88]. The reason is that these regions are more industrialized than others [88]. This result is so critical especially in Isfahan state, because a new infant milk formula plant has been established there [88]. In another survey carried out in Yazd province on raw milk, the lead content in samples were less than limit of FAO/WHO standard [90].

In China, the lead contamination in meat, eggs and milk-based products increased during last decade [91]. One of the probable reasons for this rising in other countries maybe the wide use of leaded gasoline during the recent years [88] where one of the most important sources of food lead contamination is water [92]. However, there are several factors which effect lead content in raw milk such as range of contamination of what cows graze and drink, geographical condition of pasture, distance of stable to industrial area, vehicular area, climate weather, different seasons, and type of soil [88,89].

### Other foods

Bread is the most important food of Iranian people and due to immense side effects of long-term exposure of people to contamination, being lead toxicity in daily life food seems a serious problem [93]. WHO in 1998 announced that maximum limit of lead content of bread is 0. 1 ppm [93]. Khabnadideh et al.(2004), evaluated lead concentration of collected breads from various parts of Shiraz to indicate whether bread ingredients are lead-contaminated. They found that in lead polluted area, lead level in salt and water applied by all bakeries were below the standard level (0. 05 ppm), but this level for flour samples were higher than limits [93]. This results indicate that to decrease lead contamination of bread, it is necessary to usually monitor the internal (bread ingredient, machine related to bakery) and external (distance between bakery and petrol station or to cross road) condition of bakeries [93]. In another study conducted in Finland, it was found that mean and median lead contents of all breads were 14 and 8 μg/kg. The collected samples showed a very high variation of lead contents. Also in that study, the lead content of Finnish breads was much lower than that in the late 1970s [94].

One of the sources of heavy metals and trace elements entrance to body is their release from manufacturing apparatus solving in food materials due to low pH of food [95]. pH is an important factor that effects the concentration of the cadmium and lead of the solution, because an increase of pH causes a decrease in the solubility of the lead and cadmium compounds [70].

Lemon juice and tomato paste with an average pH value of 2.3 and 4.6 are at risk of lead pollution. Poormoghaddas et al. (1998,2001) evaluated lead concentration in lemon juices and tomato paste in Isfahan city, Iran [95,96]. They found that method of preparation (non-standard apparatuses like metallic machine-made) has a significant effect in increasing the concentration of toxic elements. In hand-made juice and tomato paste, concentration of lead was in normal range where in metallic machine-made lemon juices tomato paste samples, the lead was 58% and 93% higher than normal levels [95,96].

Peanut is a kind of nut that grow in shell underground and widely eaten by people and its residue is used to richen farm animal foods [97]. Contamination of peanut, make a trouble for human and animals because it enters into human food chain through different ways. Rahbar et al. reminded in his survey that there was a quite high lead and cadmium levels in the peanut, although it depends on food habitants, geographical situation, and level of contamination environment [97].

### Medications

From a long time ago people prefer to consume herbal medicines and even doctors are in believe that herbal medicines have no side effects [98]. One of these side effects is lead toxicity which is not far from mind by rapid industrialization. Several sources of lead contamination are estimated for medical products and drugs, such some oral herbal drops which is available in markets. Asghari et al. examined 10 different oral herbal medicines in the market of Iran and found existence of heavy metals but they were below acceptable recommended intake [99]. It should be noticed that patients can be in higher risk when they use medicines for long time and their organs might not be functioning adequately to detoxify heavy metals. Of course this is not restricted to Iran and can be a concern of many countries of the world especially when trend to use herbal medicines is increasing in the world. The existence of heavy metals in herbals medicines have been confirmed in different countries [100,101]. In a study, Obi et al. (2006) evaluated existence of heavy metals in herbal drugs of Nigeria. They found that 100% of the collected samples contained elevated amounts of heavy metals. These data alert us to the possibility of heavy metals toxicity from herbal products in the public that should be studied in-depth [101].

Make up products are another source of lead exposure. Traditional eye make ups such as powder of Surma and powders of Kohl, which are used in Middle East countries, contain lead and due to the long time contact with skin and eye mucosa they can cause lead toxicity in the users [102]. In a study, Malakootian et al. (2010) evaluated the amount of lead in kohl in Kerman city. He found that mean concentration of lead in measured samples was 254.5 μg/g with range of 3.2–1219.4 μg/g. Also it was found that plant-based kohl samples had lower amount of lead in comparison to mineral-based ones [102]. Existence of lead in tooth amalgam is another catastrophic event in Iran. Amalgam has been used for tooth restoration for decades and it is still used heavily in dentistry. In an study, Mortazavi et al. (2000) evaluated substantial amounts of heavy metals and found that lead and cadmium exist in the commercial amalgam which was available in Iran at year of 2000 [103].

### Opium

Exposure to lead is usually considered only when a patient’s history points to well-known traditional sources of lead, although the incidence of lead poisoning has declined, but the presence of new forms of non-occupational poisoning poses new problems [104]. The earliest report of this strange source of lead backed to 1973 related to father and his middle aged daughter who were diagnosed as lead poisoning due to ingestion of home-made opium [105]. Additionally, acute lead poisoning as a result of self-injection of lead and opium pills, crushed and suspended in water, has been reported [105]. Inorganic lead poisoning due to intravenous or inhalation abuse of lead-contaminated heroin has been reported since 1989 [106]. Other examples include adulterated marijuana, methamphetamine and Indian herbal medicines [107-109]. In some parts of the US, illegally distilled alcohol (moonshine) is an important source of lead exposure [110].

Recently, there have been few reports about lead poisoning as a consequence of opium addiction in Iran [14,111-115]. Also, researchers reported the presence of lead in opium in the South-East Iran [116]. In the study of Salehi et al. in 2009, BLL in opium addicted patients was measured in comparison to healthy controls. The results showed that BLL in opium addicts had a range of 7.2–69.9 μg/dl with a mean of 8.6 ± 3.5 μg/dl that was significantly higher than that of controls [114]. Aghaee-Afshar et al. reported that lead existed in 10 opium samples collected from various sources with a mean concentration of 1.88 ± 0.35 PPM [116] that might be harmful in chronic consumption by addicts. Informal and often illegal laboratories refine opium into a sticky, brown paste, which is pressed into bricks and sun dried [112]. This process results in introduction of impurities such as lead into the products, but it is still unknown whether it is added to opium during the process of preparation or it is added by dealers or smugglers to increase the opium weight for more profit [104,116]. In adults, absorption of lead via the respiratory tract is the most prevalent route of opium abuse in Iran [104] since it has higher bioavailability [117] with an average absorption of approximately 40% [118]. The heat of smoking opium can affect the amount of lead absorbed in blood while other methods of consumption such as oral may have not that much effect on the opium lead and thus blood absorption of lead can be higher in these methods [119]. Also, it must be noted that several symptoms of lead poisoning are similar to that of opium abuse such as constipation, nausea, irritability, anorexia and various other neuropsychiatric symptoms. The diagnosis of lead poisoning is based on an elevated BLL, which is defined as equal to or greater than 25 μg/dL [104]. Many of toxic effects of lead is reversible if lead poisoning is identified early but high BLL and delay in treatment may lead to irreversible symptoms like motor neuron defects [111]. Unrecognized lead poisoning in drug abusers presenting with symptoms of abdominal pain can lead to misdiagnosis and unnecessary gastrointestinal evaluations or abdominal surgeries such as appendectomy [2], decreased consciousness [120], and even paralysis of four limbs [111]. Adding lead to the opium is a recently health hazard in Iran [121]. Thus, lead poisoning should be considered in patients with a history of opium abuse who present with non-specific clinical manifestations [104]. Finally, it would be noticed that substance addicts may have an elevated BLL in comparison to healthy subjects and thus screening of BLL would be helpful.

### Children exposure

Pediatric lead poisoning is still an important public health problem for millions of children in the world. In South and Central America, 33–34% of children have BLL above 10 μg/dL (0. 48 μmol/L) as compared with 7% in North America [122]. Another survey showed that one in every 20 children in the US has toxic blood levels [112]. In China, because up to 23% of populations (377 million) are children, lead exposure is still a serious public concern. Published data from this country showed that children’s BLL are higher than other developed countries due to its heavy metal pollution [123]. In another study, the mean BLL of Chinese children was 62.31 μg/L and (9. 2%) of 3,624 children’s BLL were above 100 μg/L. Taking Chinese medicinal herbs, substitutes of breast milk and puffed foods, seem the main risk factors [124].

Lead has many toxic effects on human health. In children, the most vulnerable population, mental development of children health effects is of greatest effect. Low level lead exposure can significantly induce motor dysfunctions and cognitive impairment in pediatric, especially if the exposures occurs before the age of six. Recent surveys showed that cognitive impairment is associated with BLLs < 10 mg/dL among pediatrics [125-127]. Lanphear et al. observed cognitive effects in children aged 6–16 years with BLLs < 5 mg/dL in USA [125]. Two other cross-sectional studies, in Detroit and Mexico City evaluated children at age 7 years and found an inverse relationship with BLLs <10 mg/dL and cognitive development [126,127]. Moreover, several cohort studies have provided more data that prenatal exposure to lead is associated with child cognitive development [128]. Because children and fetus are in a rapid growth course, they absorbed heavy metal in food content more than adults, so they are in exposure of greater danger than others.

Published studies indicate that children exposed to contaminated water, soils, dust, and air particulates may ingest a significant amount of lead and other toxic metals through the hand–mouth activity or the inhalation of lead dust. After banning of leaded gasoline, the US focused on paints, and the lead-based paint in old houses was considered as the main exposure resource for children lead poisoning. In the US, the main source of lead exposure in children is believed lead-based house paint and the contaminated dust [129].

There are very few studies conducted on lead exposure and its related factors in Iranian children. Faranoush et al. (2003) studied 320 students who were randomly selected in two areas in Semnan city and it was revealed that 78.8% of the children had BLLs >10 μg/dl, also, in 5% of them toxic levels of lead was observed (Pb > 20 μg/dl) [130]. In another study, it was reported that 32% of randomly selected students in a polluted district in Tehran, capital of Iran had BLLs of more than 10 μg/dl which clearly showed importance of screening test for lead poisoning in the population. Also in this study it was found that BBL in boys was 1. 6 times more than girls [36]. In another study, two groups of 7–11 years old children, from a lead mining area (Angooran, Zanjan Province, Iran) and 36 from control area, were selected to assess BLL and grown parameters such as weight and height. The mean BLLs in case group were 36.97 μg/dL which was significantly higher than controls (13. 35 μg/dL). Also there was no significant difference in growth parameters, including weight and height, in the children of two groups [131], suggesting that the BLL was not correlated with growth parameters of children in lead mining area. In another survey, the average intelligence quotient (IQ) in 64 children living in high lead area of Zanjan province (Center of Iran) was 86. 64 ± 9.68 with 40 (62. 5%) having less than normal level. In comparison in the other group, the average IQ was 91.98 ± 10.26 with 24 of the children (38. 7%) having less than normal level. The IQ of pediatric living in high-lead area was significantly lower than children living in low-lead area [132]. Moreover, Dehghan et al. evaluated BLL of children with age of 2–12 years old in Yazd city (Center of Iran) and showed that 93. 1% of children have higher BLL than standard values [133]. In another study conducted in Mashhad, East of Iran, 32 children aged 3–7 years whose parents were lead-exposed workers were randomly selected and studied. All of the children had BLL of above the standard (more than 100 μg/L). Duration of fathers’ exposure to lead at work was 9.14 ± 5.63 years. BLL was 163.81 ± 57.19 μg/L and urine lead concentration was 97 ± 48.12 mg/L. The children whose parents worked at battery plant manufacturing had higher BLL (217 mg/L) comparing to children of tile workers (151 mg/L) [134].

Although, in some countries, addition of compounds containing lead to toys has been banned in the current decade, but there are some reports which indicating lead toxicity from contaminated toys worldwide. In 2006, a child aged 4 years died of acute lead poisoning which was the first child lead-poisoning death since 2000 in the US. The autopsy revealed a heart-shaped metallic charm in the abdomen that was found to have a lead content of 99.1% [135].

In 2007, a series of recalls of pediatrics’ toys that were suspected lead contamination was issued in the US [128]. In Iran, results of some surveys showed that plastic toys and other PVC products manufactured for children in some area are contaminated with lead [136]. In Iran comparing to other countries, studies on lead exposure and lead toxicity in the children are too few. In fact sources of lead exposure in Iranian children are unknown. In recognizing that there is no safe BLL for pediatric and chelating agents have limited value in decreasing the harmful effect of lead poisoning or even cost-benefit. The government should control or eliminate lead hazards in children’s environment before they are exposed.

### Pre- and post-natal exposure

There is a strong relation between umbilical cord and maternal BLL which proves the transfer of lead from mother to fetus [128]. Golmohammadi et al. (2007) evaluated lead concentration of specimens of maternal blood, new born, cord blood, and colostrums in polluted area of Tehran and compared with non-polluted areas. Their data revealed an association between mean concentrations in blood lead of mothers and newborns and between mean concentrations of colostrums lead and newborn BLL in both area. The lead concentration of mother blood, newborn cord blood and colostrums in polluted area were significantly higher than non-polluted area. The mean BLL of mothers, cord blood of newborns and colostrums were 7.6 ± 4.1, 5.9 ± 3, and 4.2 ± 2.5 μg/dl, respectively in the non-polluted area and they were 9.1 ± 8.4, 6.5 ± 5.2, and 5.8 ± 5.5 μg/dl, respectively, in the polluted area [137]. Moreover, Pourjafari et al. (2007) evaluated the fetal deaths rate among progenies of workers at two high risk occupations (lead mine and dye-houses) of Hamadan city (West of Iran) and the results were compared with general population. The rates of abortions plus stillbirths among their wives’ pregnancies were 13.15 and 13.30%, respectively. Fetal death rates were significantly higher than general population that suggests the idea that long-term genetic consequences occur following working in lead mine and dye-houses [138].

In addition, Vigeh et al. (2010) conducted a study to clarify the effects of lead on fetal premature rupture of the membranes (PROM). They measured BLL in 332 women with age range of 16–35 years, during their early pregnancy period. They found that BLL of PROM deliveries were significantly higher than non-PROM deliveries (4.61 ± 2.37 g/dl versus 3.69 ± 1.85 g/dl) and suggested that lead can increase the risk of PROM in pregnant women even with mean BLL less than 5 g/dl [139].

Moreover, Vige et al. (2011) studied the effect of lead on occurrence of preterm labor. The BLL of mothers who delivered preterm babies was significantly higher than that of those who delivered full-term babies (4.46 ± 1.86 versus 3.43 ± 1.22|μg/dl). This suggests that adverse pregnancy outcomes may occur at BLL even below the current acceptable level [140].

Norouzi et al. (2010) conducted a study to determine concentration of lead in the milk of women living in the vicinity of a metal smelter area. Their results showed that mean level of lead in milk nulliparous women was significantly higher than multiparous women (70.64 versus 23.73 g/l). Also, they found that milk lead level of women with age of 24 or less was significantly higher than age greater than 24 years old [141].

In addition, cohort surveys indicate that prenatal exposure is associated with pediatric cognitive development [128]. Hu et al. (2006) evaluated fetal lead toxicity during trimesters of pregnancy as predictors of infant neurodevelopment. They revealed that exposure to lead during the first trimester may have a greater effect on adverse neurodevelopment later in life than the second or third trimesters [142]. The Mexico City Prospective Lead survey [143] showed that higher maternal BLL at third trimester of pregnancy, especially around week 28, was correlated to reduction of intellectual child development. Wasserman et al. [144] studied the impact of pre and postnatal lead exposure to early intelligence and found that pre and postnatal exposures to lead that occur during the first 7 years of life are independently accompanied with small reduction in later IQ scores [144].

## Conclusion

Several metal chelators can be used to prevent lead poisoning after occurrence of exposure or can save life in persons with very high BLL but none of them are suitable in reducing lead burden in chronic lead exposure [128]. Moreover, chelators are not always available in all countries or if available they are too expensive [145] and are not included by health insurance companies and most importantly they have limited value in decreasing the sequel of lead poisoning. Also some clinical trials demonstrated no developmental benefit in the group that received succimer after 3 and 7 years treatment [146,147]. These findings highlight the importance of undertaking further precautions and designing programs to prevent lead contamination. Identification of sources, elimination or control of sources, and monitoring environmental exposures and hazards can be used to prevent lead poisoning. Hopefully Department of Public Health and Environment from World Health Organization has made special attention to update guidelines on the prevention and management of lead poisoning started since 2011. The first meeting was held 11–13 July 2011 at WHO Geneva, Switzerland where the corresponding author of this paper attended as an adviser. The meeting tried to recognize world problem of lead poisoning country by country to reach a global conclusion about protocols and measures to prevent lead poisoning. One of the conclusions of the meeting was to ask all countries to extend their studies to recognize source of lead exposure and extent of toxicity in their people by general screening and finally conducting systematic reviews country by country. Taken collectively, authors recommend the following actions to be considered in Iran.

1) Full prohibition of use of leaded gasoline and alternating with naturally obtained ethanol as a clean fuel.

2) Authorities should introduce measures for assessing and controlling occupational exposure to lead and exposure of workers’ families. People who work with lead should be taught about the hazards of lead and how to minimize their own exposure as well as exposure to their families.

3) Authorities should conduct systematic country-wide studies to identify, document and map the important environmental sources of lead exposure in their populations.

4) Authorities should work towards elimination of the non-essential use of lead in household and consumer products (including paint, toys, solder in food cans, lead batteries, cosmetics, traditional medicines or remedies, ceramics used in connection with food, cosmetics, etc.). Where water is supplied through lead pipes, these pipes should be replaced with safer materials or water filters should be used to reduce the lead content of the water. In homes where painted with leaded paint, the paint should either be removed or stabilized using appropriate safe measures.

5) Public health authorities should establish and implement a BLL screening program in populations known or suspected to be at risk of lead exposure. Reference laboratories can be set up to regularly measurement of lead in biological and environmental samples. In this regard, setting national limits for concentration of lead in air, water, food and soil that are based on the protection of human health in vulnerable populations should be taken into priority. Special attention to children would need establishing national children's environmental health centers to provide effective and rapid diagnosis, treatment and increased awareness of lead poisoning and other environmental threats.

6) The removal, remediation and/or replacement of lead-contaminated soil in communities where lead contamination is high should be considered by authorities.

7) Where there is a significant risk of exposure to lead from environmental and other sources, parents and pregnant women should be taught about the hazards of lead and how to reduce or prevent exposure. Generally, education of all public at lead-polluted area should be taken into priority through media.

As a final point, this review confirms that chronic lead toxicity should be concerned for Iranian population because sources of lead pollution in air, waters, soil, food, and etc. exist. Although many studies have been conducted on environmental lead contamination in Iran but there are very few studies evaluated current status of lead exposure and toxicity in the Iranian children and pregnant women. Screening of lead exposures especially in children and prevention strategies should be a priority for Iranian authorities. We recommend identify sources, eliminate or control sources, and monitor environmental exposures and hazards to prevent lead poisoning.

## Competing interests

The authors declare that they have no competing interests.

## Authors’ contributions

PK and OM collected data and drafted the manuscript. MA gave the idea, designed and supervised the study, and edited the manuscript. All authors contributed equally. All authors read and approved the final manuscript.

## Author’s information

Mohammad Abdollahi is an Adviser to the World Health Organization (WHO) for providing WHO guidelines on the prevention and management of lead poisoning. He is doing his best to integrate identification of lead toxicity in human and its hazards to the environment.

## References

[B1] Malekirad AA Oryan S Fani A Babapor V Hashemi M Baeeri M Bayrami Z Abdollahi M Study on clinical and biochemical toxicity biomarkers in a zinc-lead mine workers Toxicol Ind Health 2010 26 6 331 337 10.1177/0748233710365697 20371635

[B2] Mohammadi S Mehrparvar A Aghilinejad M Appendectomy due to lead poisoning: a case-report J Occup Med Toxicol 2008 17 3 23 18928540 10.1186/1745-6673-3-23 PMC2575197

[B3] Abdollahi M Sadeghi Mojarad A Jalali N Lead toxicity in employees of a paint factory MJIRI 1996 10 203 206

[B4] Azizi MH Azizi F Lead poisoning in the world and Iran Int J Occup Environ Med 2010 1 81 87 23022790

[B5] Karimooy HN Mood MB Hosseini M Shadmanfar S Effects of occupational lead exposure on renal and nervous system of workers of traditional tile factories in Mashhad (northeast of Iran) Toxicol Ind Health 2010 26 9 633 638 10.1177/0748233710377774 20630982

[B6] Landrigan PJ Todd AC Lead poisoning West J Med 1994 161 2 153 159 7941534 PMC1022528

[B7] Balali-Mood M Shademanfar S Rastegar Moghadam J Afshari R Namaei Ghassemi M Allah Nemati H Keramati MR Neghabian J Balali-Mood B Zare G Occupational lead poisoning in workers of traditional tile factories in Mashhad, Northeast of Iran Int J Occup Environ Med 2010 1 1 23022779

[B8] Eslami S Hajizadeh Moghaddam A Jafari N Nabavi SF Nabavi SM Ebrahimzadeh MA Trace element level in different tissues of Rutilus frisii kutum collected from Tajan River, Iran Biol Trace Elem Res 2011 143 2 965 973 10.1007/s12011-010-8885-9 20978865

[B9] Abdollahi M Ebrahimi-Mehr M Nikfar S Jalali N Monitoring of lead poisoning in simple workers of a copying center by flame atomic absorption spectroscopy MJIRI 1996 10 69 72

[B10] Staudinger KC Roth VS Occupational lead poisoning Am Fam Physician 1998 57 4 719 726 731–2 9490995

[B11] Mañay N Cousillas AZ Alvarez C Heller T Lead contamination in Uruguay: the “La Teja” neighborhood case Rev Environ Contam Toxicol 2008 195 93 115 10.1007/978-0-387-77030-7_4 18418955

[B12] Paoliello MM De Capitani EM Occupational and environmental human lead exposure in Brazil Environ Res 2007 103 2 288 297 10.1016/j.envres.2006.06.013 16919621

[B13] Abdollahi M Shohrati M Nikfar S Jalali N Monitoring of lead poisoning in bus drivers of Tehran Irn J Med Sci 1995 20 29 33

[B14] Masoodi M Zali MR Ehsani-Ardakani MJ Mohammad-Alizadeh AH Aiassofi K Aghazade R Shavakhi A Somi MH Antikchi MH Yazdani S Abdominal pain due to lead-contaminated opium: a new source of inorganic lead poisoning in Iran Arch Iran Med 2006 9 72 75 16649384

[B15] Kalantari S Khoshi AH Mohebbi MR Fooladsaz K Investigation of blood lead levels and its toxicity in workers of zinc melting factory of Dandi, Zanjan, Iran J Zanjan Univ Med Sci Health Serv 2009 17 66 79 86

[B16] Pirsaraei SR Lead exposure and hair lead level of workers in a lead refinery industry in Iran Indian J Occup Environ Med 2007 11 1 6 8 10.4103/0019-5278.32457 21957365 PMC3168112

[B17] Tabrizizadeh M Boozarjomehri F Akhavan Karbasi MH Maziar F Evaluation of the relationship between blood lead level and prevalence of oral complication in Koushk lead mine workers, Yazd province J Dent Tehran Univ Med Sci 2006 19 1 91 99

[B18] Aminpour MR Barkhordari A Ehrampoush MH Hakimian AM Blood lead levels in workers at Kooshk lead and zinc mine J Shahid Sadoughi Univ Med Sci Health Serv 2008 16 2 24 30

[B19] Malekirad AA Fani A Abdollahi M Oryan S Babapour V Shariatzade SMA Davodi M Blood-urine and cognitive –mental parameters in mine workers exposed to lead and zinc AMUJ 2011 13 4 106 113

[B20] Shahrabi J Dorosti AR Study of blood lead levels, hemoglobin & plasma ascorbic acid in a car company welders Iran J Occup Health 2006 3 1–2 50 55

[B21] Meshkinian A Asilian H Nazmara Sh Shahtaheri DJ Determination of Lead in the environment and in the urban services workers in Tehran municipality district J Sch Public Health Inst Public Health Res 2003 1 3 31 40

[B22] Yartireh HA Determination of blood and urine lead level among workers of Kermanshah refinery in 1994 Sci Med J 2001 31 60 65

[B23] Herman DS Geraldine M Venkatesh T Evaluation, diagnosis, and treatment of lead poisoning in a patient with occupational lead exposure: a case presentation J Occup Med Toxicol 2007 2 7 10.1186/1745-6673-2-7 17718907 PMC2000868

[B24] Shiri R Ansari M Ranta M Falah-Hassani K Lead poisoning and recurrent abdominal pain Ind Health 2007 45 3 494 496 10.2486/indhealth.45.494 17634699

[B25] Mirsattari SG Urine lead levels in service station attendants exposed to tetraethyl lead J Res Med Sci 2001 6 3 151 154

[B26] Yaghmaie B Faghihi Zarandi A Bazrafshani M Arjomand Tajaddini A Study of lead concentration in the air of gasoline station of Kerman city J Kerman Univ Med Sci 1995 2 2 66 70

[B27] Keramati MR Nemai Ghasemi M Balali-Mood M Correlation between iron deficiency and lead intoxication in the workers of a car battery manufacturer J Birjand Univ Med Sci 2009 16 1 51 58

[B28] Ibrahim AS Latif AH Adult lead poisoning from a herbal medicine Saudi Med J 2002 23 5 591 593 12070589

[B29] Leung AO Duzgoren-Aydin NS Wong H Heavy metals concentrations of surface dust from e-waste recycling and its human health implications in southeast China Environ Sci Technol 2008 42 7 2674 2680 1 10.1021/es071873x 18505015

[B30] Centers for Disease Control and Prevention Preventing lead poisoning in young children 2005 Centers for Disease Control, Atlanta, GA

[B31] Abdollahi M Zadparvar L Ayatollahi B Baradaran M Nikfar S Hastaie P Khorasani R Hazard from carbon monoxide poisoning for bus drivers in Tehran, Iran Bull Environ Contam Toxicol 1998 61 2 210 5 10.1007/s001289900750 9702358

[B32] Kebriaeezadeh A Abdollahi M Sharifzadeh M Mostaghasi R Lead levels in the inhabitants of Tehran city districts Pajouhandeh 1997 2 5 72 67

[B33] Farzin L Amiri M Shams H Ahmadi Faghih MA Moassesi ME Blood levels of lead, cadmium, and mercury in residents of Tehran Biol Trace Elem Res 2008 123 1–3 14 26 18278442 10.1007/s12011-008-8106-y

[B34] Naddafi K Ehrampoush MH Jafari R Nabizadeh M Younesian M Complete evaluation of suspended air particles and their composition in the central area of Yazd city J Shahid Sadoughi Univ Med Sci Health Serv 2008 16 1 21 25

[B35] Farahmandkia Z Mehrasbi MR Sekhawatju MS Hasanalizadeh AS Ramezanzadeh Z Study of heavy metals in the atmospheric deposition in Zanjan, Iran Iran J Health Environ 2010 2 4 240 249

[B36] Zaman T Hosseinzadeh H Lead poisoning in a highly polluted district of Tehran in high school children Iran J Pediatr 1999 4 207 212

[B37] Chuturkova R Iossifova Y Clark S Decrease in ambient air lead concentrations in Varna, Bulgaria, associated with the introduction of unleaded gasoline Ann Agric Environ Med 2010 17 2 259 261 21186768

[B38] WHO WHO Guidelines for drinkingwater quality 2006 World Health Organization, Geneva 35 38

[B39] Karbasi M Karbasi E Saremi A Ghorbani Zade Kharazi H Determination of heavy metals concentration in drinking water resources of Aleshtar in 2009 YAFT-E 2010 12 1 65 70

[B40] Fertmann R Hentschel S Dengler D Janssen U Lommel A Lead exposure by drinking water: an epidemiologial study in Hamburg, Germany Int J Hyg Environ Health 2004 207 3 235 244 10.1078/1438-4639-00285 15330391

[B41] Renner R Out of plumb: when water treatment causes lead contamination Environ Health Perspect 2009 117 12 A542 A547 20049189 10.1289/ehp.117-a542 PMC2799485

[B42] Watt GC Britton A Gilmour WH Moore MR Murray GD Robertson SJ Womersley J Is lead in tap water still a public health problem? An observational study in Glasgow BMJ 1996 313 7063 979 981 19 10.1136/bmj.313.7063.979 8892418 PMC2352296

[B43] Zietz BP Lass J Suchenwirth R Dunkelberg H Lead in drinking water as a public health challenge Environ Health Perspect 2010 118 4 A154 A155 10.1289/ehp.1001979 20368134 PMC2854742

[B44] Haider T Haider M Wruss W Sommer R Kundi M Lead in drinking water of Vienna in comparison to other European countries and accordance with recent guidelines Int J Hyg Environ Health 2002 205 5 399 403 10.1078/1438-4639-00164 12173540

[B45] Karimpour M Shariat MA A study of heavy metals in drinking water network in Hamadan city in 1994 Sci J Hamadan Univ Med Sci Health Serv 2007 7 17 47 44

[B46] Savari J Jaafarzadeh N Hassani AH Shams Khoramabadi GH Heavy metals leakage and corrosion potential in Ahvaz drinking water distribution network Water Wastewater J 2008 18 64 16 24

[B47] Shahmansouri MR Poormoghadas H Shams Khorramabadi GH A study of Leakage of trace metals from corrosion of the municipal drinking water distribution system J Res Med Sci 2003 8 3 34 30

[B48] Tashauoei HR Hajian Nejad M Amin MM Karakani F A study on leakage of heavy metals from the PVC and polypropylene pipes used in the water distribution system in Isfahan Health Syst Res 2010 6 3 373 382

[B49] Ebrahimi A Ehrampoush MH Ghaneian MT Davoudi M Hashemi H Behzadi S The survey chemical quality of ground water in the vicinity of sanitary landfill of Yazd in 2008 Health Syst Res 2010 6 1048 1056

[B50] Ebrahimi A Amin MM Hashemi H Foladifard R Vahiddastjerdi M A survey of groundwater chemical quality in Sajad Zarinshahr Health Syst Res 2011 6 918 926

[B51] Legg TM Zheng Y Simone B Radloff KA Mladenov N González A Knights D Siu HC Rahman MM Ahmed KM McKnight DM Nemergut DR Carbon, metals, and grain size correlate with bacterial community structure in sediments of a high arsenic aquifer Front Microbiol 2012 3 82 22470368 10.3389/fmicb.2012.00082 PMC3311048

[B52] Nazemi S Khosravi A A study of heavy metals in soil, water and vegetables Knowledge Health 2011 5 4 27 31

[B53] Mohammadian M Nouri J Afshari N Nassiri J Nourani M Investigation of heavy metal concentrations in the water wells close to Zanjan zinc and lead smelting plant Iran J Health Environ 2008 1 51 56

[B54] Zafarzadeh A The determination of water chemical quality of cisterns in rural areas of Golestan province J Gorgan Univ Med Sci 2006 8 51 54

[B55] Yaziz MI Gunting H Sapari N Ghazali AW Variations in rainwater quality from roof catchments Water Res 1989 23 761 765 10.1016/0043-1354(89)90211-X

[B56] Rabito FA Iqbal S Perry S Arroyave W Rice JC Environmental lead after Hurricane Katrina: implications for future populations Environ Health Perspect 2012 120 2 180 184 22052045 10.1289/ehp.1103774 PMC3279443

[B57] Farsam H Zand N A preliminary study of lead deposition on plant leaves in Tehran Iran J Publ Health 1991 20 1–4 27 34

[B58] Jalali M Khanlari ZV Enviromental contamination of Zn, Cd, Ni, Cu and Pb from industrial areas in Hamadan Province, western Iran Environ Geol 2008 55 1537 1543 10.1007/s00254-007-1103-1

[B59] Parizanganeh A Hajisoltani P Zamani A Assessment of heavy metal pollution in surficial soils surrounding Zinc Industrial Complex in Zanjan-Iran Procedia Environ Sci 2010 2 162 166

[B60] Chehregani A Noori M Yazdi HL Phytoremediation of heavy-metal-polluted soils: screening for new accumulator plants in Angouran mine (Iran) and evaluation of removal ability Ecotoxicol Environ Saf 2009 72 5 1349 1353 10.1016/j.ecoenv.2009.02.012 19386362

[B61] Masoudi SN Ghajar Sepanlou M Bahmanyar MA Distribution of lead, cadmium, copper and zinc in roadside soil of Sari-Ghaemshahr road, Iran Afr J Agric Res 2012 7 2 198 204

[B62] Piron-Frenet M Bureau F Pineau A Lead accumulation in surface roadside soil: its relationships to traffic density and meteorological parameters Sci Total Environ 1994 144 297 304 10.1016/0048-9697(94)90449-9

[B63] Ozkan M Gurkan R Ozkan A Akcay M Determination of manganese and lead in roadside soil samples by FAAS with ultrasound assisted leaching J Anal Chem 2005 60 469 474 10.1007/s10809-005-0121-y

[B64] Parizanganeh A Lakhan VC Jalalian H A geochemical and statistical approach for assessing heavy metal pollution in sediments from the southern Caspian coast Int J Environ Sci Tech 2007 4 3 351 358

[B65] Shahriari A Determination of heavy metals (Cd, Cr, Pb, Ni)in edible tissues of Lutjans Coccineus and Tigeratooh Croaker in Persian Gulf-2003 J Gorgan Univ Med Sci 2005 7 16 67 65

[B66] Tabari S Saravi SS Bandany GA Dehghan A Shokrzadeh M Heavy metals (Zn, Pb, Cd and Cr) in fish, water and sediments sampled form Southern Caspian Sea, Iran Toxicol Ind Health 2010 26 10 649 656 10.1177/0748233710377777 20639278

[B67] Ebrahimi M Taherianfard M Concentration of four heavy metals (cadmium, lead, mercury, and arsenic) in organs of two cyprinid fish (Cyprinus carpio and Capoeta sp.) from the Kor River (Iran) Environ Monit Assess 2010 168 575 585 10.1007/s10661-009-1135-y 19711190

[B68] Shokrzadeh M Ebadi AG Heidari R Zare S Measurement of lead, cadmium and chromium in five species of mostconsumed fish in Caspian sea Int J Biol Biotech 2004 1 673 675

[B69] Dorafshan S Heyrati FP Spawning induction in Kutum Rutilus frisii kutum (Kamenskii, 1901) using carp pituitary extract or GnRH analogue combined with metoclopramide Aquacult Res 2006 37 751 755 10.1111/j.1365-2109.2006.01488.x

[B70] Bakhtiarian A Gholipour M Ghazi-Khansar M Lead and cadmium of Korbal rice in Northern Iran Iran J Publ Health 2001 30 3–4 129 132

[B71] National Research Council Oil in the sea. Inputs fates and effects 1985 National Academy Press, Washington DC

[B72] Price ARG Shepparrd CRC The Gulf: past, present, and possible future states Marine Pollut Bull 1991 22 222 227 10.1016/0025-326X(91)90914-E

[B73] Ashraf W Accumulation of heavy metals in kidney and heart tissues of Epinephelus microdon fish from The Arabian Gulf Environ Monit Assess 2005 101 1–3 311 316 15736888 10.1007/s10661-005-0298-4

[B74] Raissy M Ansari M Rahimi E Mercury, arsenic, cadmium and lead in lobster (Panulirus homarus) from the Persian Gulf Toxicol Ind Health 2011 27 7 655 659 10.1177/0748233710395346 21357635

[B75] Pourmoghaddas H Shahryari A The concentration of lead, chromium, cadmium, nickel and mercury in three speices of consuming fishes of Isfahan city Health Syst Res 2010 6 1 30 35

[B76] Malakootian M Yaghmaeian K Meserghani M Mahvi AH Daneshpajouh M Determination of Pb, Cd, Cr and Ni concentration in Imported Indian Rice to Iran Iran J Health Environ 2011 4 1 77 84

[B77] Jahedkhaniki GR Zazoli MA Cadmium and lead contents in rice (Oryza sativa) in the North of Iran Int J Agr Biol 2005 7 6 1026 1029

[B78] Lin HT Wong SS Li GC Heavy metal content of rice and Shellfish in Taiwan J Food Drug Anal 2004 12 167 174

[B79] Shakerian A Rahimi E Ahmadi M Cadmium and lead content in several brands of rice grains (Oryza sativa) in central Iran Toxicol Ind Health 2012 Epub ahead of print 10.1177/0748233711430979 22258626

[B80] Zazouli MA Shokrzadeh M Izanloo H Fathi S Cadmium content in rice and its daily intake in Ghaemshahr region of Iran Afr J Biotechnol 2008 7 3686 3689

[B81] Malakootian M Aboli M Ehrampoosh M Determination of lead level in Lettuce in Kerman Tolooe Behdasht 2009 8 1–2 62 67

[B82] Ebadi AG Zare S Mahdavi M Babaee M Study and measurement of Pb, Cd, Cr and Zn in green leaf of tea cultivated in Gillan province of Iran Pak J Nutr 2005 4 270 272

[B83] Maleki A Zarasvand MA Heavy metals in selected edible vegetables and estimation of their daily intake in Sanandaj, Iran Southeast Asian J Trop Med Public Health 2008 39 2 335 340 18564723

[B84] Jahangir-Touyserkani A Tavakolian F Valaie N Determination of the lead, cadmium, copper in the vegetables, grown in south part of Tehran Pajouhandeh 2004 9 38 105 108

[B85] Asadi M Bazargan N Survey of heavy metal in the waste water of a canal after irrigation of vegetable farms in south of Tehran Iran J Public Health 1994 23 4–1 44 35

[B86] Malakootian M Mesreghani M Danesh Pazhoo M A survey on Pb, Cr, Ni and Cu concentration in Tehran consumed black tea Sci J Rafsanjan Univ Med Sci Health Serv 2011 10 138 143

[B87] Matsuura H Hokura A Katsuki F Itoh A Haraguchi H Multielement determination and speciation of major-to-trace elements in black tea leaves by ICP-AES and ICP-MS with the aid of size exclusion chromatography Anal Sci 2001 17 3 391 398 10.2116/analsci.17.391 11990615

[B88] Tajkarimi M Ahmadi-Faghih M Poursoltani H Salahnejad A Motallebi AA Mahdavi H Lead residue levels in raw milk from different regions of Iran Food Control 2008 19 5 495 498 10.1016/j.foodcont.2007.05.015

[B89] Caggiano R Sabia S D’Emilio M Macchiato M Anastasio A Ragosta M Paino S Metal levels in fodder, milk, dairy products, and tissues sampled in ovine farms of Southern Italy Environ Res 2005 99 1 48 57 10.1016/j.envres.2004.11.002 16053927

[B90] Yasaei-Mehrgrdy GHR Ezzatpanah H Yasini-Ardakani SA Dadfarnia SH Assessment of lead and cadmium levels in raw milk from various regions of Yazd province Food Tech Nutr 2010 7 35 42

[B91] Wang M Wang Z Ran L Han H Wang Y Yang D Study on food contaminants monitoring in China during 2000–2001 Wei Sheng Yan Jiu 2003 32 4 322 326 14535092

[B92] Renner R Out of plumb: when water treatment causes lead contamination Environ Health Perspect 2009 117 12 A542 7 20049189 10.1289/ehp.117-a542 PMC2799485

[B93] Khabnadideh S Mokhtarifard A Namavar-Jahromi B Malekpour MB Lead detection in bread ingredients of fifth district of Shiraz city in 2000 Hakim Res J 2004 7 17 21

[B94] Tahvonen R Kumpulainen J Lead and cadmium contents in Finnish breads Food Addit Contam 1994 11 5 621 631 10.1080/02652039409374262 7835475

[B95] Poormoghaddas H Javadi I Eslamieh R Cadmium, chromium, mercury and lead concentration in lemon joices J Res Med Sci 1998 3 114 118

[B96] Poormoghaddas H Javadi I Eslamieh R Study of the toxic trace elements cadmium, lead, and mercury in tomato paste in Isfahan city Sci J Hamedan Univ Med Sci Health Serv 2002 9 40 46

[B97] Rahbar N Nazari Z Level of lead and cadmium in peanut KAUMSJ (FEYZ) 2004 7 71 77

[B98] Eisenberg DM Advising patients who seek alternative medical therapies Ann Intern Med 1997 127 1 61 69 9214254 10.7326/0003-4819-127-1-199707010-00010

[B99] Asghari G Palizban AA Tolue-Ghamar Z Adeli F Contamination of cadmium, lead and mercury on Iranian herbal medicines Tabriz J Pharm Sci 2008 1 1 8

[B100] Abou-Arab AAK Soliman Kawther M El Tantawy ME Ismail Badeaa R Naguib K Quantity estimation of some contaminants in commonly used medicinal plants in the Egyptian market Food Chem 1999 67 357 363 10.1016/S0308-8146(99)00082-5

[B101] Obi E Akunyili DN Ekpo B Orisakwe OE Heavy metal hazards of Nigerian herbal remedies Sci Total Environ 2006 369 1–3 35 41 16759683 10.1016/j.scitotenv.2006.04.024

[B102] Malakootian M Pourshaaban Mazandarany M Hossaini H Lead levels in powders of surma (Kohl) used in Kerman J Kerman Univ Med Sci 2010 17 167 174

[B103] Mortazavi VS Fathi MH Tooth restoration with Amalgam; treatment or tragedy J Dent School 2000 18 32 40

[B104] Soltaninejad K Fluckiger A Shadnia SH Opium addiction and lead poisoing J Subst Use 2011 16 3 208 212 10.3109/14659891.2010.545860

[B105] Chino M Moriyama K Saito H Morn T The amount of heavy metal derived from domestic sources in Japan Water Air Soil Pollut 1991 57-58 1 57 829–837

[B106] Antonini G Palmieri G Millefiorini E Spagnoli LG Millefiorini M Lead poisoning during heroin addiction Ital J Neurol Sci 1989 10 1 105 108 10.1007/BF02333882 2925342

[B107] Dunbabin DW Tallis GA Popplewell PY Lee RA Lead poisoning from Indian herbal medicine (Ayurveda) Med J Aust 1992 157 11–12 835 836 1454025 10.5694/j.1326-5377.1992.tb141305.x

[B108] Norton RL Burton BT McGirr J Blood lead of intravenous drug users J Toxicol Clin Toxicol 1996 34 4 425 430 10.3109/15563659609013813 8699557

[B109] Busse F Omidi L Timper K Leichtle A Windgassen M Kluge E Stumvoll M Lead poisoning due to adulterated marijuana N Engl J Med 2008 358 15 1641 1642 10.1056/NEJMc0707784 18403778

[B110] Morgan BW Barnes L Parramore CS Kaufmann RB Elevated blood lead levels associated with the consumption of moonshine among emergency department patients in Atlanta, Georgia Ann Emerg Med 2003 42 3 351 358 10.1016/S0196-0644(03)00491-8 12944887

[B111] Beigmohammadi MT Aghdashi M Najafi A Mojtahedzadeh M Karvandian K Quadriplegia due to lead-contaminated opium–case report Middle East J Anesthesiol 2008 19 6 1411 1416 18942257

[B112] Jalili M Azizkhani R Lead toxicity resulting from chronic ingestion of opium West J Emerg Med 2009 10 4 244 246 20046241 PMC2791725

[B113] Afshari R Emadzadeh A Case report on adulterated opium-induced severe lead toxicity Drug Chem Toxicol 2010 33 1 48 49 10.3109/01480540903127340 20001217

[B114] Salehi H Sayadi AR Tashakori M Yazdandoost R Soltanpoor N Sadeghi H Aghaee-Afshar M Comparison of serum lead level in oral opium addicts with healthy control group Arch Iran Med 2009 12 555 558 19877747

[B115] Fatemi R Jafarzadeh F Moosavi S Afshar Amin F Acute lead poisoning in an opium user: a case report GHFBB 2008 1 3 139 142

[B116] Aghaee-Afshar M Khazaeli P Behnam B Rezazadehkermani M Ashraf-Ganjooei N Presence of lead in opium Arch Iran Med 2008 11 5 553 554 18759525

[B117] Mahaffey KR Quantities of lead producing health effects in humans: sources and bioavailability Environ Health Perspect 1977 19 285 295 908307 10.1289/ehp.7719285 PMC1637386

[B118] Froutan H Kashefi Zadeh A Kalani M Andrabi Y Lead toxicity: a probable cause of abdominal pain in drug abusers MJIRI 2011 25 16 20

[B119] Hayatbakhsh Abbasi MM Ansari M Shahesmaeili A Qaraie A Lead serum levels in opium-dependent individuals Addic Health 2009 1 2 106 110 PMC3905488 24494092

[B120] Moharari RS Khajavi MR Panahkhahi M Mojtahedzadeh M Najafi A Loss of consciousness secondary to lead poisoning–case reports Middle East J Anesthesiol 2009 20 3 453 455 19950744

[B121] Karrari P Mehrpour O Balali-Mood M Iranian crystal: a misunderstanding of the crystal-meth J Res Med Sci 2012 17 2 96 97 23264800 PMC3525044

[B122] Jones RL Homa DM Meyer PA Brody DJ Caldwell KL Pirkle JL Brown MJ Trends in blood lead levels and blood lead testing among US children aged 1 to 5 years, 1988–2004 Pediatrics 2009 123 3 e376 e385 10.1542/peds.2007-3608 19254973

[B123] Wang S Zhang J Blood lead levels in children, China Environ Res 2006 101 3 412 418 10.1016/j.envres.2005.11.007 16442092

[B124] Shi H Jiang YM Li JY Liu F Wang H Yu F Yang H Environmental lead exposure among children in Chengdu, China, 2007–2009 Biol Trace Elem Res 2011 143 1 97 102 10.1007/s12011-010-8849-0 20890733

[B125] Lanphear BP Dietrich K Auinger P Cox C Cognitive deficits associated with blood lead concentrations <10 microg/dL in US children andadolescents Public Health Rep 2000 115 6 521 529 10.1093/phr/115.6.521 11354334 PMC1308622

[B126] Chiodo LM Jacobson SW Jacobson JL Neurodevelopmental effects of postnatal lead exposure at very low levels Neurotoxicol Teratol 2004 26 3 359 371 10.1016/j.ntt.2004.01.010 15113598

[B127] Kordas K Canfield RL López P Rosado JL Vargas GG Cebrián ME Rico JA Ronquillo D Stoltzfus RJ Deficits in cognitive function and achievement in Mexican first-graders with low blood leadconcentrations Environ Res 2006 100 3 371 386 10.1016/j.envres.2005.07.007 16169549

[B128] Meyer PA Brown MJ Falk H Global approach to reducing lead exposure and poisoning Mutat Res 2008 659 1–2 166 175 18436472 10.1016/j.mrrev.2008.03.003

[B129] Lin GZ Peng RF Chen Q Wu ZG Du L Lead in housing paints: an exposure source still not taken seriously for children lead poisoning in China Environ Res 2009 109 1 1 5 10.1016/j.envres.2008.09.003 18976991

[B130] Faranoush M Malek M Ghorbani R Rahbar M Safaei Z Study of the blood lead levels and related factors in the 6–11 years old children in Semnan Koomesh 2003 4 3 79 86

[B131] Mahram M Mousavinasab N Dinmohammadi H Soroush S Sarkhosh F Effect of living in lead mining area on growth Indian J Pediatr 2007 74 6 555 559 10.1007/s12098-007-0107-x 17595498

[B132] Mahram M Intelligence quotient level of the children living high lead areas in Zanjan province Behbood 2004 7 4 36 42

[B133] Dehghan L Ghane A Study of the blood lead levels in the 2–12 years old children in Yazd. [MD dissertation] 2000 Faculty of Medicine, Yazd Univ Med Sci, Iran,Yazd 70 85

[B134] Deldar K Nazemi E Balali-Mood M Emami SA Mohammadpour AH Tafaghodi M Afshari R Effect of Corandrum sativum L. extract on lead excretion in 3–7 year old children J Birjand Univ Med Sci Health Serv 2008 15 3 11 19

[B135] CDC Death of a child after ingestion of a metallic charm--Minnesota MMWR Morb Mortal Wkly Rep 2006 55 340 341 16572103

[B136] Pourmoghaddas H Pishkar AR Kavehzade FM Perecentage of toxic trace elements; Pb, Cr, and Cd in certain plastic toys, Isfahan city J Shahid Sadoughi Univ Med Sci Health Serv 2006 14 59 64

[B137] Golmohammadi T Ansari M Nikzamir A Safary R Elahi S The effect of maternal and fetal lead concentration on birth weight: polluted versus non-polluted areas of Iran Tehran Univ Med J 2007 65 8 74 78

[B138] Pourjafari H Rabiee S Pourjafari B Fetal deaths and sex ratio among progenies of workers at lead mine and dye-houses Genet Third Millennium 2007 5 2 1057 1060

[B139] Vigeh M Yokoyama K Shinohara A Afshinrokh M Yunesian M Early pregnancy blood lead levels and the risk of premature rupture of the membranes Reprod Toxicol 2010 30 3 477 80 10.1016/j.reprotox.2010.05.007 20576532

[B140] Vigeh M Yokoyama K Seyedaghamiri Z Shinohara A Matsukawa T Chiba M Yunesian M Blood lead at currently acceptable levels may cause preterm labour Occup Environ Med 2011 68 3 231 4 10.1136/oem.2009.050419 20798002

[B141] Norouzi E Bahramifar N Ghasempouri SM Determination concentration of lead in breast in lactating women in region industrial zarinshahr and effect on infant J Isfahan Med School 2010 28 112 640 646

[B142] Hu H Téllez-Rojo MM Bellinger D Smith D Ettinger AS Lamadrid-Figueroa H Schwartz J Schnaas L Mercado-García A Hernández-Avila M Fetal lead exposure at each stage of pregnancy as a predictor of infant mental development Environ Health Perspect 2006 114 11 1730 5 17107860 10.1289/ehp.9067 PMC1665421

[B143] Schnaas L Rothenberg SJ Flores MF Martinez S Hernandez C Osorio E Velasco SR Perroni E Reduced intellectual development in children with prenatal lead exposure Environ Health Perspect 2006 114 5 791 7 16675439 10.1289/ehp.8552 PMC1459938

[B144] Wasserman GA Liu X Popovac D Factor-Litvak P Kline J Waternaux C LoIacono N Graziano JH The Yugoslavia Prospective Lead Study: contributions of prenatal and postnatal lead exposure toearly intelligence Neurotoxicol Teratol 2000 22 6 811 8 10.1016/S0892-0362(00)00106-9 11120386

[B145] Nikfar S Khatibi M Abdollahiasl A Abdollahi M Cost and utilization study of antidotes: an Iranian experience Int J Pharmacol 2011 7 46 49

[B146] Rogan WJ Dietrich KN Ware JH Dockery DW Salganik M Radcliffe J Jones RL Ragan NB Chisolm JJ Rhoads GG The effect of chelation therapy with succimer on neuropsychological development in children exposed to lead N Engl J Med 2001 344 19 1421 6 10.1056/NEJM200105103441902 11346806

[B147] Dietrich KN Ware JH Salganik M Radcliffe J Rogan WJ Rhoads GG Fay ME Effect of chelation therapy on the neuropsychological and behavioral development of lead-exposed children after school entry Pediatrics 2004 114 1 19 26 10.1542/peds.114.1.19 15231903

